# Pharmacological primary and secondary cardiovascular prevention among diabetic patients in a multiethnic general practice population: still room for improvements

**DOI:** 10.1186/1472-6963-13-182

**Published:** 2013-05-20

**Authors:** Anh T Tran, Jørund Straand, Ingvild Dalen, Kåre I Birkeland, Tor Claudi, John G Cooper, Haakon E Meyer, Anne K Jenum

**Affiliations:** 1 Department of General Practice, Institute of Health and Society, University of Oslo, Oslo, Norway; 2 Faculty of Medicine, University of Oslo, Oslo, Norway; 3 Department of endocrinology, obesity and preventive medicine, University of Oslo, Oslo, Norway; 4 Department of Medicine, Nordland Hospital, Bodø, Norway; 5 Department of Medicine, Stavanger University Hospital, Stavanger, Norway; 6 Department of Community Medicine, Institute of Health and Society, University of Oslo, Oslo, Norway; 7 Division of Epidemiology, Norwegian Institute of Public Health, Oslo, Norway; 8 Oslo and Akershus University College of Applied Sciences, Oslo, Norway

**Keywords:** Type 2 diabetes, CVD prevention, Ethnicity, General practice

## Abstract

**Background:**

Ethnic minority groups have higher prevalence of cardiovascular disease (CVD) and type 2 diabetes mellitus (T2DM). We assessed general practitioners’ (GPs’) performance with respect to the pharmacological prevention of CVD in patients with T2DM from different ethnic backgrounds in Oslo.

**Methods:**

Of 1653 T2DM patients cared for by 49 GPs in 2005, 380 had a diagnosis of CVD. Ethnicity was categorized as Norwegian, South Asian and other. Risk factor levels, medication use, achievement of treatment targets (HbA1c ≤ 7.5%, systolic blood pressure (SBP) ≤ 140 mmHg, total cholesterol/HDL-cholesterol < 4) and therapeutic intensity (number of drugs targeting each risk factor) were recorded. Chi-square, Wald tests and multiple linear regression analyses were used.

**Results:**

Of the 1273 patients receiving primary prevention, 1.5% had their Hb1Ac, 4.8% SBP and 12.7% lipids levels above treatment thresholds without relevant prescriptions. Among patients on pharmacological therapy, 66% reached the HbA1c, 62% SBP and 62% lipid target. Proportions not achieving the HbA1c target were 26% in Norwegians, 38% in South Asians and 29% in others (p = 0.008). Proportions not achieving the SBP target were 42% in Norwegians, 22% in South Asians and 25% in others (p ≤ 0.001). Of those not achieving the HbA1c and SBP targets, 43% and 35% respectively, used only one agent.

In secondary prevention, 0.8% of the patients had their HbA1c, 0.5% SBP and 7.4% lipid levels above treatment thresholds without relevant prescriptions. Among patients on pharmacological therapy, 65% reached the HbA1c, 64% SBP and 66% lipid target. Proportions not achieving the HbA1c target were 26% in Norwegians, 47% in South Asians and 40% in others (p = 0.03). Proportions not achieving the SBP target were 36% in Norwegians, 22% in South Asians and 56% in others (p = 0.050). Of those not achieving HbA1c and SBP targets, 49% and 21% respectively, were on mono-therapy.

**Conclusions:**

Norwegian GPs comply reasonably well with guidelines for pharmacological prevention of CVD in T2DM patients across ethnic groups. However, lipid-lowering therapy was generally underused, and the achievement of treatment targets for HbA1c in ethnic minorities and for BP in Norwegians could be improved.

## Background

Cardiovascular disease (CVD), in particular coronary heart disease (CHD) and cerebro-vascular disease, are the major causes of morbidity and mortality in patients with diabetes [1,2]. In Europe, ethnic groups with origin from Asia and Africa have a higher prevalence of type 2 diabetes mellitus (T2DM) [3,4], higher age-adjusted diabetes mortality rates [5] and standardised CVD mortality rates [6] than the majority population.

In several Western countries, improvements in risk factor levels and better treatment for CHD have contributed to reduced CVD mortality in both the general population [7-12] and in individuals with diabetes [13-17]. Data from the UK reveal that these improvements have not to the same extent benefitted ethnic minorities like South Asians [18].

Because patients with highest risk also gain most from interventions, they should be identified and prioritized by their general practitioners (GPs) [19]. Pharmacological therapy to prevent CVD in individual patients should therefore be based on estimated absolute risk for future CVD [19,20]. Intensive interventions targeting multiple risk factors to prevent CVD in T2DM patients with ethnic minority backgrounds are needed to reduce ethnic disparities in long term health outcomes [21-23]. To our knowledge, only two diabetes specific risk algorithms, those developed by The United Kingdom Prospective Diabetes Study (UKPDS) Group [24] and the New Zealand Diabetes Cohort Study Group [25], include ethnicity as a risk factor. However, guidelines must be tailored to fit the individual. Too intensive glucose-lowering therapy (e.g. aiming at HbA1c < 6%) may increase mortality in patients with previous cardiovascular events [26] or in elderly patients with a long duration of diabetes [27]. Similarly, intensive anti-hypertensive therapy, aiming at systolic blood pressure (SBP) well below 130 mmHg has also been questioned due to increased risk for hypotension and adverse drug reactions [28]. Moreover, interventions to reduce blood pressure below 120 mmHg have not been shown to reduce cardiovascular risk [29].

Compared with the majority population, ethnic minority groups in Norway have a higher prevalence of self-reported CVD and diabetes [30], increased susceptibility for diabetes for a given level of adiposity [31], and are on average younger at the time of T2DM diagnosis [32]. Despite receiving more intensive glucose-lowering therapy, they also have poorer glycaemic control [32]. Regardless of ethnicity, important processes of care measures are comparable, but only one in four of T2DM patients in all ethnic groups, receiving care in general practice, reach all national treatment targets related to HbA1c, BP and lipid levels [32].

The aim of this study was to explore GPs’ adherence to the guidelines for pharmacological primary or secondary CVD prevention in T2DM patients [33,34], achievements of treatment targets, the intensity of treatment, potential overtreatment and to estimate the 10-year risk for CHD in Norwegian and South Asian patients without known CVD.

## Methods

### Design, setting and participants

Cross-sectional data from electronic medical records (EMRs) from 11 practices (49 GPs’ with 58857 listed patients) from multiethnic parts of Eastern Oslo were used [33]. A specially designed data programme was used to identify patients with diabetes and to capture predefined data from the EMR from the years 2003–2005. Data regarding HbA1c, SBP, DBP, microalbuminuria, body weight and foot examinations were from 2005, for eye examinations from 2004 or 2005, and for s-cholesterol and smoking habits from 2003 to 2005. A total of 2064 patients with a diagnosis of diabetes were identified. As our aim was to explore GPs’ adherence to guidelines for T2DM, we excluded T1DM patients (n = 103), T2DM patients with two or more diabetes related hospital visits the previous 12 months (n = 178) as they also had incomplete information of current medication, those with less than six months of follow-up or who had moved or were deceased (n = 128), or who had incomplete information about the country of birth (n = 2), leaving 1653 T2DM patients cared for by their GPs to be included in the present study. The study was approved by the Regional Ethics Committee West, the Directorate for Health and the Data Inspectorate.

### Variables

Patients who according to the EMR had a diagnosis of angina pectoris, previous myocardial infarction, stroke or transitory ischemic attack, or intermittent claudication, were categorized as having CVD and thus requiring secondary prevention (SP) to reduce their risk of new events. All other patients were considered to require primary prevention (PP) of CVD.

According to the Norwegian national general practice diabetes guidelines from 2005 [33,34], diabetes patients without CVD should have anti-hypertensive therapy if their BP > 140/90 mmHg, and lipid lowering therapy if their total cholesterol/HDL-cholesterol ratio > 5.0 and they had at least one additional risk factor (current anti-hypertensive therapy, smoking, microalbumiuria or a family history of premature CVD). For diabetes patients with CVD, the corresponding treatment thresholds were BP > 140/90 mmHg and total cholesterol/HDL-cholesterol ratio ≥ 4.0 irrespective of other risk factors. Treatment targets were: HbA1c ≤ 7.5%, SBP ≤ 140 mmHg, diastolic blood pressure (DBP) ≤ 85 mmHg, and total cholesterol/HDL-cholesterol ratio < 4.0 [33,34].

Age, gender, recorded measurements of CVD risk factors, patient’s age when diabetes was diagnosed, disease duration, and prescription data were captured [32]. For HbA1c, SBP, DBP and lipids, the most recent results were selected.

Prescription of glucose-lowering therapy (anti-diabetic agents, insulin or any combinations), anti-hypertensive (angiotensin converting enzyme inhibitors, calcium channel blockers, alfa blockers, beta blockers, angiotensin II receptor antagonists, diuretics, or any combinations), and lipid-lowering therapy (statins) were dichotomized as “yes” versus “no” for all three therapy groups. The intensity of treatment was categorized by numbers of agents used in combination to target elevated BP, and to lower blood glucose. Because too intensive treatment may put patients at increased risk for side effects like hypoglycaemia and hypotension, we identified patients on glucose-lowering therapy with HbA1c < 6.0% [27,35] and those on anti-hypertensive therapy with SBP < 130 mmHg or DBP < 65 mmHg [28].

Ethnicity was based on self-reported country of birth as recorded in the EMR, and categorized as: Norwegians (including about 2% from other Scandinavian countries or Western Europe/North America), South Asians (Pakistanis, Sri Lankans and Indians), others (from other low- and middle income countries).

To estimate individual 10-year absolute risk for CHD for patients without prior CVD in the two largest ethnic groups (Norwegians and South Asians), we used the UKPDS Risk Engine version 2 [24], which includes age, gender, diabetes duration, HbA1c, SBP, total cholesterol, HDL-cholesterol, smoking status (never, past or current smoker).

### Statistical analyses

Chi- squared, one-way ANOVAs, T-tests and Wald tests were used to test differences between proportions and means in the groups. Because HbA1c values were highly skewed, they were log-transformed and presented as geometric means (estimates with 95% confidence intervals, transformed back to the original scale). Generalized linear models were applied to estimate means for the CVD risk factors, the proportions receiving pharmacological therapy, and the proportions not reaching treatment targets, all adjusted for age and gender. The geometric mean values for HbA1c were also adjusted for diabetes duration.

HbA1c was recorded in 95%, BP in 91%, total cholesterol in 94% and smoking habits in 59% of all patients, but the complete set of variables to be used in the UKPDS risk engine was available for only 54% of Norwegian and 43% of South Asian patients. We therefore used multiple imputation techniques for individuals with incomplete data as this is the recommended procedure to limit bias due to missing data when values are missed at random [36]. Age, gender, ethnicity, BMI, glucose-lowering therapy, anti-hypertensive therapy and lipid-lowering therapy were used as predictors for the imputed values and five imputed datasets were created. Multiple regression models were applied to estimate age- and gender adjusted 10-year mean risk for cases with complete data and for all cases after imputation and pooling of the original and imputed data sets.

Two-sided tests were used and p-values ≤ 0.05 were considered statistically significant. The analyses were performed with SPSS 19.0 for Windows and Stata 12.

## Results

Of the 1653 included patients with T2DM, 1273 (77%) had no CVD diagnoses (PP group), whereas 380 had one or more CVD diagnoses (SP group). South Asians were youngest both in the PP and SP group, being on average 13 and 18 years younger than Norwegians at the time of diabetes diagnosis (Table 1). In the PP group the prevalence of current smoking was lowest among South Asians (p = 0.001).

**Table 1:** Characteristics of 1653 patients with type 2 diabetes receiving pharmacological prevention of CVD by ethnicity

**Characteristics**^**a**^	**Valid cases**	**Norwegians**	**South Asians**	**Others**^**b**^	**P**^**c**^
**Primary prevention of CVD (n, %)**	1273 (100.0)	830 (65.2)	265 (20.8)	178 (14.0)	
Males, %	601	47.2	55.7	49.4	0.737
Age, years, mean ( 95% CI)	1273	64.3 (63.4-65.2)	51.0 (49.9-52.1)	54.2 (52.6-55.7)	<0.001
Age at diagnosis of diabetes, years, mean (95% CI)	1180	58.0 (57.1-58.9)	44.6 (43.4-45.7)	48.2 (46.4-50.0)	<0.001
Diabetes duration, years, mean (95% CI)	1180	6.2 (5.8-6.6)	6.1 (5.4-6.8)	5.1 (4.4-5.8)	0.077
Current smoker, % (95% CI)	742	25.7 (22.6-28.9)	10.8 (8.7-13.2)	21.9 (19.0-25.0)	0.001
**Secondary prevention of CVD (n, %)**	380 (100.0)	299 (78.7)	57 (15.0)	24 (6.3)	
Males, %	227	57.5	68.4	66.7	0.238
Age, years, mean, (95% CI)	380	72.4 (71.2-73.6)	58.2 (55.8-60.6)	65.5 (61.2-69.9)	<0.001
Age at diagnosis of diabetes, years, mean, (95% CI)	346	64.7 (63.3-66.0)	46.5 (43.8-49.3)	54.7 (50.1-59.3)	<0.001
Diabetes duration, years, mean (95% CI)	346	7.5 (6.7-8.2)	11.5 (9.4-13.5)	9.5 (7.0-11.9)	0.001
Current smoker, % (95% CI)	240	22.4 (17.7-28.2)	12.1 (8.5-16.7)	20.5 (15.8-26.0)	0.405

^a^ CVD: cardiovascular disease (i.e. angina pectoris or myocardial infarction or stroke or intermittent claudication).

^b^ Patients from other regions.

^c^ p-values. Chi-square test was applied to compare proportion between ethnic groups. One-way ANOVAs were applied to compare mean age, age at diagnosis of diabetes and diabetes duration between ethnic groups.

### Primary prevention of CVD

Among the 1273 T2DM patients without established CVD, 95% had their HbA1c, 90% SBP, 94% total cholesterol and 58% smoking habits recorded. Differences between ethnic groups for these processes of care were minor and significant only for BP-measurements which were less prevalent among South Asians. In total, 950 (75%) received pharmacological glucose-lowering, 700 (55%) anti-hypertensive and 401 (32%) lipid-lowering therapy. Of those on pharmacological therapy 66% reached the HbA1c, 62% SBP and 62% lipid target.

Age- and gender-adjusted mean values for SBP, DBP, HDL-cholesterol and geometric means for HbA1c (also adjusted for diabetes duration), differed significantly between ethnic groups (Table 2). In accordance with risk factor levels, glucose-lowering therapy was prescribed more often and anti-hypertensives less often to ethnic minority patients compared with Norwegians, but the proportion not achieving treatment targets still differed between ethnic groups. Although lipid-lowering therapy was prescribed less often to minority patients, the proportion not reaching the lipid target did not differ between the ethnic groups (Table 2).

**Table 2:** Primary cardiovascular prevention by ethnicity (n=1273): risk factors, pharmacological intervention and proportion not achieving targets

**Characteristics**^**a**^	**Valid cases**	**Norwegians (n=830)**	**South Asians (n=265)**	**Others**^**b **^**(n=178)**	**P**^**c**^
**Age- and gender adjusted last available measure of risk factors for CVD (mean, 95% CI)**					
HbA1c, %	1205	6.9 (6.78-6.94)	7.4 (7.24-7.53)	7.2 (7.01-7.37)	<0.001
SBP, mmHg	1142	137.6 (136.4-138.7)	128.6 (126.4-130.8)	131.0 (128.5-133.5)	<0.001
DBP, mmHg,	1141	80.3 (79.6-80.9)	76.5 (75.3-77.8)	77.7 (76.2-79.0)	<0.001
Total chol, mmol/L	1198	5.3 (5.19-5.33)	5.1 (4.95-5.21)	5.3 (5.14-5.45)	0.049
LDL-chol, mmol/L	793	3.2 (3.09-3.24)	3.2 (3.01-3.31)	3.2 (3.02-3.37)	0.935
HDL-chol, mmol/L	1150	1.4 (1.35-1.40)	1.3 (1.22-1.32)	1.3 (1.29-1.40)	0.001
Total chol/HDL-chol	1150	4.0 (3.96-4.14)	4.2 (4.02-4.34)	4.2 (4.03-4.41)	0.181
**Age- and gender adjusted proportion receiving pharmacological therapy (%, 95% CI)**					
Glucose lowering	1273	71.6 (68.4-74.7)	81.4 (76.6-86.3)	78.7 (72.7-84.8)	0.003
Anti-hypertensive	1273	62.2 (58.7-65.7)	41.2 (34.7-47.7)	42.2 (34.7-49.8)	<0.001
Lipid-lowering	1273	35.4 (32.0-38.8)	28.4 (22.7-34.0)	16.2 (10.9-21.6)	<0.001
**Age- and gender adjusted proportions not achieving treatment targets among patients receiving pharmacological therapy (%, 95% CI)**					
HbA1c >7.5%	899	25.7 (21.2-30.2)	38.2 (30.5-45.9)	29.2 (21.2-37.3)	0.008
SBP > 140 mmHg	672	41.9 (37.5-46.4)	21.6 (12.2-31.0)	24.5 (14.0-35.1)	<0.001
DBP > 85 mmHg	671	25.4 (21.6-29.2)	25.1 (15.9 -34.2)	25.1 (14.7-35.3)	0.996
Total Chol/HDL-chol ≥ 4.0	382	37.3 (31.4-43.1)	33.0 (22.0- 44.1)	48.1 (29.5-66.8)	0.382

^a^ Multiple linear regression was used to estimate means and multiple logistic regression was used to estimate proportions in the groups adjusted for age and gender. HbA1c was additionally adjusted for diabetes duration. SBP: systolic blood pressure. DBP: diastolic blood pressure. HbA1c was log-transformed before applying the multiple regression model and the geometric means is presented.

Glucose- lowering therapy: prescription of anti-diabetic agents, insulin or any combinations. Anti-hypertensive therapy: prescription of one or combination of several agents. Lipid-lowering therapy: prescription of statins.

^b^ Patients from other regions than Western Europe/ North America and SA.

^c^ p-values. Wald tests were applied to test for differences in means and proportions between the groups adjusted for age and gender.

Treatment was more intense in those not achieving targets for hyperglycaemia and SBP (Table 3), but 43.4% and 35.3% respectively used only one agent. Among the 82 patients on glucose-lowering therapy despite having HbA1c < 6%, 6 patients were > 75 years, 4 had a diabetes duration > 10 years and 17 received two or three glucose-lowering agents (Table 4). Among the 154 patients on anti-hypertensive therapy having SBP < 130 mmHg, 56 were receiving two or more agents.

**Table 3:** Intensity of pharmacological therapy in T2DM patients receiving cardiovascular prevention by CVD status

**Characteristics**^**a**^	**Valid cases**	**Intensity of therapy**		
		**One agent**	**Two agents**	**Three or more agents**
**Primary prevention of CVD**				
**Proportions of patients on glucose lowering therapy achieving and not achieving treatment target (%, 95% CI)**				
HbA1c ≤ 7.5%	595	63.9 (59.9-67.6)	34.6 (30.9-38.5)	1.5 (0.8-2.9)
HbA1c > 7.5%	304	43.4 (38.0-49.9)	51.0 (45.4-56.6)	5.6 (3.5-8.8)
P < 0.001^b^				
**Proportions of patients on anti-hypertensive therapy achieving and not achieving treatment target (%, 95% CI)**				
SBP ≤ 140 mmHg	414	43.7 (39.0-48.5)	29.0 (24.8-33.5)	27.3 (23.2-31.8)
SBP > 140 mmHg	258	35.3 (29.7-41.3)	28.3 (23.1-34.1)	36.4 (30.7-42.3)
P=0.028^b^				
DBP ≤ 85 mmHg	499	41.1 (36.9-45.5)	28.7 (24.9-32.8)	30.3 (26.4-34.4)
DBP > 85 mmHg	172	39.0 (32.0-46.4)	28.5 (22.2-35.7)	32.6 (26.0-39.9)
P=0.834^b^				
**Secondary prevention of CVD**				
**Proportions of patients on glucose lowering therapy achieving and not achieving treatment target (%, 95% CI)**				
HbA1c ≤ 7.5%	183	58.5 (51.2-65.4)	37.7 (31.4-44.9)	3.8 (1.7-7.8)
HbA1c > 7.5%	97	48.5 (38.8-58.3)	47.4 (37.8-57.3)	4.1 (1.3-10.5)
P=0.269^b^				
**Proportions of patients on anti-hypertensive therapy achieving and not achieving treatment target (%, 95% CI)**				
SBP ≤ 140 mmHg	205	31.7 (25.7-38.4)	30.2 (24.4-36.9)	38.0 (31.7-44.9)
SBP > 140 mmHg	114	21.1 (14.5-29.5)	34.2 (26.1-43.3)	44.7 (35.9-53.9)
P=0.125^b^				
DBP ≤ 85 mmHg	268	28.4 (23.3-34.0)	31.7 (26.4-37.5)	39.9 (34.2-45.9)
DBP > 85 mmHg	51	25.5 (15.4-39.0)	31.4 (20.3-45.1)	43.1 (30.5-56.7)
P=0.888^b^				

^a^ CVD: cardiovascular disease. Treatment target for glucose-lowering therapy: HbA1c ≤ 7.5%, for antihypertensive therapy: systolic blood pressure (SBP) ≤ 140 mmHg and diastolic blood pressure (DBP) ≤ 85 mmHg.

^b^ P-values. Chi-square tests were applied to compare proportions of patients receiving one, two or three agents and between those achieving and not achieving the specific treatment targets.

**Table 4:** Potential overtreatment in T2DM patients receiving pharmacological cardiovascular prevention by CVD status and ethnicity

**Characteristics**^**a**^	**Total**	**Norwegians**	**South Asians**	**Other**	**p**^**b**^
**Primary cardiovascular prevention in 1273 patients without known CVD**					
**Proportions of patients on glucose lowering therapy, n/N (%)**					
HbA1c < 6.0%	82/899 (9.1)	62/563 (11.0)	7/202 (3.5)	13/134 (9.7)	0.006
**Proportions of patients on anti-hypertensive therapy, n/N (%)**					
SBP < 130 mmHg	154/672 (22.9)	98/514 (19.1)	36/90 (40.0)	20/68 (29.4)	<0.001
DBP < 65 mmHg	25/671 (3.7)	18/514 (3.5)	6/89 (6.7)	1/68 (1.5)	0.193
**Secondary cardiovascular prevention in 380 patients with known CVD**					
**Proportions of patients on glucose lowering therapy, n/N (%)**					
HbA1c < 6.0%	25/280 (8.9)	24/209 (11.5)	1/52 (1.9)	0/19 (0.0)	0.035
**Proportions of patients on anti-hypertensive therapy, n/N (%)**					
SBP < 130 mmHg	76/319 (23.8)	55/262 (21.0)	17/40 (42.5)	4/17 (23.5)	0.012
DBP < 65 mmHg	26/319 (8.2)	16/262 (6.1)	6/40 (15.0)	4/17 (23.5)	0.009

^a^ CVD: cardiovascular disease. Potential overtreatment with glucose-lowering therapy if HbA1c < 6.0%, with antihypertensive therapy if systolic blood pressure (SBP) < 130 mmHg or diastolic blood pressure (DBP) < 65 mmHg. n: number of patients on pharmacological therapy with HbA1c < 6.0%, SPB < 130 mmHg or DBP < 65 mmHg, N: valid cases.

^**b**^ P-values. Chi-square tests were applied to compare proportions between ethnic groups.

Among patients without glucose-lowering therapy, 5% had HbA1c > 7.5% and among those without anti-hypertensive therapy, 11% had SBP > 140 mmHg. Among patients without lipid-lowering therapy, 19% had total cholesterol/HDL-cholesterol ratio > 5. Of the total PP group, 1.5% had hyperglycaemia, 4.8% hypertension and 12.7% dyslipidemia without relevant prescriptions. No ethnic differences were observed for non-prescription of preventive pharmacological treatment.

Risk estimation for CHD by the UKPDS engine based on the 54% of Norwegian and 43% of South Asian patients with complete datasets, revealed comparable age- and gender-adjusted 10-year absolute risk (95% confidence interval) for CHD between the groups (South Asians: 19.0(17.5-20.5)%, Norwegians: 17.2(16.5-17.9)%). Patients with (n = 560) and without (n = 535) complete data for CVD risk estimations were comparable for age at diagnosis, SBP, total cholesterol, and HDL-cholesterol, but differed for age (60 years vs. 62 years, p = 0.027) diabetes duration (5.8 years vs. 6.6 years, p = 0.027), HbA1c (7.0% vs. 7.2%, p = 0.001) and proportions with smoking habits recorded (100% vs. 20%, p<0.001). After imputation and pooling of datasets, the age- and gender 10-year risk estimates for CHD increased for both groups (South Asians: 20.9 (19.7-22.1)%, Norwegians 19.9 (18.7-21.1)%, but ethnic differences remained insignificant.

### Secondary prevention of CVD

In the SP group, the proportions of patients with recorded CVD risk factors were comparable between ethnic groups. Pharmacological therapy was more intensive than in the PP group (glucose-lowering drugs: 78%, anti-hypertensives: 89%, lipid-lowering drugs: 64%). Among those on pharmacological therapy, 65% reached the HbA1c, 64% SBP and 66% lipid target, comparable with the PP group.

HbA1c, DBP, HDL- cholesterol and total cholesterol/HDL-cholesterol ratio differed between the ethnic groups (Table 5). Compared with the PP group, the ethnic differences for HbA1c were larger in the SP group. For patients on pharmacological therapy not achieving treatment targets, a similar pattern to that found in the PP group was observed for the ethnic groups (Table 5).

**Table 5:** Secondary cardiovascular prevention by ethnicity (n=380): risk factors, pharmacological intervention and proportion not achieving targets

**Characteristics**^**a**^	**Valid cases**	**Norwegians (n=299)**	**South Asians (n=57)**	**Others**^**b **^**(n=24)**	**P**^**c**^
**Age- and gender adjusted last available measure of risk factors for CVD (mean, 95% CI)**					
HbA1c, %	360	7.0 (6.84-7.13)	7.9 (7.48-8.28)	7.5 (6.96-8.08)	<0.001
SBP, mmHg	356	135.5 (136.3-140.7)	134.3 (128.8-139.8)	138.9 (131–146.7)	0.370
DBP, mmHg	356	78.7 (77.6-79.8)	73.7 (70.9-76.4)	72.5 (68.6-76.4)	<0.001
Total chol, mmol/L	349	4.7 (4.58-4.83)	4.6 (4.30-4.87)	4.8 (4.40-5.25)	0.596
LDL-chol, mmol/L	231	2.7 (2.59-2.88)	2.6 (2.30-2.95)	2.8 (2.21-3.34)	0.817
HDL-chol, mmol/L	332	1.3 (1.27-1.36)	1.2 (1.07-1.26)	1.2 (1.10-1.37)	0.013
Total chol/HDL-chol	332	3.7 (3.60-3.88)	4.1 (3.80-4.41)	4.1 (3.67-4.58)	0.049
**Age- and gender adjusted proportion receiving pharmacological therapy (%, 95% CI)**					
Glucose lowering	380	73.8 (68.5-79.0)	96.5 (91.7-101.3)	87.9 (75.0-100.9)	<0.001
Anti-hypertensive	380	91.1 (87.8-94.4)	86.6 (77.4-95.9)	80.7 (64.9-96.5)	0.345
Lipid-lowering	380	66.3(60.4-72.1)	60.7(46.1-75.3)	67.5(47.5-87.5)	0.781
**Age- and gender adjusted proportions not achieving treatment targets among patients receiving pharmacological therapy (%, 95% CI)**					
HbA1c >7.5%	280	26.0 (19.4-32.6)	47.2 (31.4- 63.0)	39.6 (16.3-62.9)	0.031
SBP > 140 mmHg	319	35.5 (29.5-41.5)	22.3 ( 7.7 – 36.8)	56.1 (32.2 – 80.0)	0.050
DBP > 85 mmHg	319	16.5 (12.0-21.1)	12.2 (2.3-22.1)	11.6 (−3.6-26.7)	0.645
Total Chol/HDL chol ≥ 4.0	226	31.8 (24.8-39.0)	43.8 (27.6-59.9)	28.5 (7.1 – 50.0)	0.378

^a^ Multiple linear regression was used to estimate means and multiple logistic regression was used to estimate proportions in the groups adjusted for age and gender. HbA1c was additionally adjusted for diabetes duration. SBP: systolic blood pressure. DBP: diastolic blood pressure. HbA1c was log-transformed before applying the multiple regression model and the geometric means is presented.

Glucose- lowering therapy: prescription of anti-diabetic agents, insulin or any combinations. Anti-hypertensive therapy: prescription of one or combination of several agents. Lipid-lowering therapy: prescription of statins.

^b^ Patients from other regions than Western Europe/ North America and SA.

^c^ p-values. Wald tests were applied to test for differences in means and proportions between the groups adjusted for age and gender.

Among the 25 patients on glucose-lowering therapy with a HbA1c < 6.0%, 12 were > 75 years, 12 had a diabetes duration > 10 years and 10 received two or three glucose-lowering agents (Table 4). Of the 76 patients on antihypertensive therapy with SBP < 130 mmHg, 21 used two or more anti-hypertensives.

Among patients without glucose-lowering therapy, 4% had HbA1c > 7.5% while 5% of those without anti-hypertensive therapy had SBP > 140 mmHg. Among patients without lipid-lowering therapy, 21% had total cholesterol/HDL-cholesterol ratio ≥ 4. Of the total SP group, 0.8% had hyperglycaemia, 0.5% hypertensjon and 7.4% dyslipidemia without relevant prescriptions. No ethnic differences were observed for these proportions.

## Discussion

Our study adds to a sparse literature on primary and secondary prevention of CVD in T2DM patients of different ethnic origin in general practice. Norwegian GPs comply reasonably well with the guidelines with respect to which patients should be prescribed glucose-lowering and anti-hypertensive therapy across different ethnic groups of patients, but lipid lowering therapy was relatively underused. However, for those on pharmacological treatment, both in the PP and SP group, at least one in three did not reach specified treatment targets, and a significant proportion of these patients used only one drug to lower their HbA1c or BP. There seems to be a potential to safely improve the achievement of treatment targets, especially regarding glucose-lowering therapy in South Asians and anti-hypertensive therapy in Norwegians, as very few seemed to be at risk of potentially adverse effects due to overtreatment of hyperglycemia or hypertension, especially in the PP group.

### Strengths and limitations

Our study population is probably representative for the population in suburban areas in Eastern Oslo [32]. Other strengths are the detailed recording of prescriptions, HBA1c, BP and lipid values in EMRs together with physician based diagnosis of diabetes and CVD.

When sampling the data, our focus was on GPs’ adherence to guidelines, and patients receiving specialist care were excluded. This represents a limitation in relation to the total diabetes population. Furthermore, we lack potentially important information regarding side effects of pharmacological therapy, lifestyle intervention and language barriers [37] or health literacy [38] which may influence adherence with prescribed medication [39].

Missing data limit the validity of our results regarding the estimated risk for future CHD. Multiple imputation of missing values may, however, limit this problem if data are missed at random. Estimates after imputation therefore probably reflect the whole study population [36]. The shorter diabetes duration and a better glycaemic control in patients with complete data for risk estimation may explain why the risk estimates increased after imputation. Estimated CHD risks should nevertheless be interpreted with caution due to this limitation.

### Adherence to the guidelines

Norwegian GPs performed good quality diabetes care with respect to recommended measurements of HbA1c, SBP and total cholesterol. Our findings here are comparable with those reported from a British general practice study [40]. Although most patients with risk factor levels above treatment thresholds received pharmacological therapy according to guidelines, a significant proportion did not achieve treatment targets despite receiving more intensive treatment compared with those who reached targets for HbA1c and SBP. On the other hand, our results may suggest that patients with higher age, longer diabetes duration and poorer glycaemic control are more likely to miss regular appointments. The GPs should pay particular attention to these patients.

In spite of receiving more intensive glucose-lowering therapy, the ethnic minority groups still had higher HbA1c than the Norwegians, both in PP and SP. This finding is in accordance with previous findings from British general practice [41,42] and may reflect lower age at diagnosis [42,43], more pronounced insulin resistance, language barriers or poorer adherence with prescribed therapy [44,45]. In Norwegian T2DM patients, under-treatment of hypertension or non-adherence with prescribed medication may represent a challenge.

However, a patient’s acceptance of using an intensive and complex pharmacological regimen for CVD prevention must be considered. The patients’ preferences, cultural factors, skepticism towards polypharmacy [37,39,46] may all influence GPs’ prescribing practice and patients’ adherence to prescribed therapy and thereby also health outcomes.

Furthermore, the GPs have to take into account additional costs in terms of adverse drug reactions and possible interactions in polypharmacy, without added clinical benefits [28,47]. This is even more relevant as the latest version of the Norwegian guidelines promote even lower threshold levels for HbA1c (≤ 7.0%) and BP (135/80 mmHg), if not otherwise contraindicated [48]. Hypotension may represent barriers for doctors and patients. However, a recent systematic review and meta-analysis concluded that intensive anti-hypertensive therapy may provide greater protection against CVD events and micro-vascular complications, not least in patients with diabetes [49]. Furthermore, very tight glycaemic control must be individualized and the possible benefit of a reduction of microvascular complications must be weighed against the possibility of hypoglycemia [50] and increased mortality, especially in elderly patients with CVD and long diabetes duration [26]. In this study we have no information about side effects of therapy and the GPs strategies for shared decision-making with their patients.

At the time of our study, GPs seemed to focus more on glucose-lowering and anti-hypertensive than on lipid-lowering therapy. Since we collected our data, prescription of newer glucose-lowering agents and statins in the general population has increased [51]. Statins have a modest effect on HDL-cholesterol levels, other strategies for lipid therapy may be considered, especially for South Asians where HDL-cholesterol seems to be particularly important [52].

### CHD risk estimation

The age- and gender adjusted 10-year risk for CHD were lower than corresponding estimates from the US (22.5%) [53] and in Australian general practice (20.3%) [54]. This may relate to our exclusion of patients receiving specialist care, who may be at higher risk for CVD. The UKPDS Risk Engine is considered to be a useful tool to identify individuals with T2DM at high risk for CVD in order to target preventive therapy, but may not provide equally valid absolute risk estimates in different populations [55,56]. We do not have cause-specific mortality statistics for different ethnic minority groups in Norway. However, lower rates of hypertension and smoking in South Asians may balance out the effect of higher HBA1c compared with Norwegians [53].

## Conclusions

GPs adhered to guidelines and prescribed pharmacological therapy for the primary and secondary prevention of CVD in most T2DM patients with risk factor levels above treatment thresholds in all ethnic groups. However, more intensive pharmacological therapy may be justified for the relatively large proportion of patients on pharmacological therapy that does not reach treatment targets. This relates in particular to lipid-lowering therapy for all ethnic groups, antihypertensive therapy for Norwegians and glucose-lowering therapy in ethnic minorities. Reaching treatment targets for HbA1c in South Asians is challenging both in primary and secondary prevention. Future clinical guidelines for the primary and secondary prevention of CVD in patients with T2DM should take into account the role of ethnicity. Updated risk estimates based on observed CVD morbidity- and mortality rates for the major ethnic groups are warranted.

## Competing interests

The authors declare that they have no competing interests.

## Authors’ contributions

The data collection was conceived and designed by AKJ, TC, and JGC. ATT and ID performed the analysis. All authors participated in discussing the results. ATT wrote the first draft of the manuscript and all authors commented on the drafts and approved the final version.

## Pre-publication history

The pre-publication history for this paper can be accessed here:

http://www.biomedcentral.com/1472-6963/13/182/prepub
